# Predictors of Cardiovascular Symptoms Among Long COVID Patients: Data from the Polish Long COVID Cardiovascular (PoLoCOV-CVD) Study

**DOI:** 10.3390/jcm14030956

**Published:** 2025-02-02

**Authors:** Joanna Kapusta, Siamala Sinnadurai, Mateusz Babicki, Żaneta Kałuzińska-Kołat, Wouter C. Meijers, Damian Kołat, Olivier C. Manintveld, Piotr Jankowski, Michał Chudzik

**Affiliations:** 1 Department of Internal Diseases, Rehabilitation and Physical Medicine, Medical University of Lodz, 90-647 Lodz, Poland; joanna.kapusta@umed.lodz.pl; 2 Cardiovascular Institute, Thorax Center, Department of Cardiology, Erasmus MC, 3015 GD Rotterdam, The Netherlands; w.meijers@erasmusmc.nl (W.C.M.); o.manintveld@erasmusmc.nl (O.C.M.); 3 Department of Pathology & Clinical Bioinformatics, Erasmus MC, 3015 GD Rotterdam, The Netherlands; 4 Department of Epidemiology and Health Promotion at the School of Public Health Medical Center for Postgraduate Education, 01-826 Warsaw, Poland; piotr.jankowski@cmkp.edu.pl; 5 Department of Family Medicine, Wroclaw Medical University, 51-141 Wroclaw, Poland; ma.babicki@gmail.com; 6 Department of Biomedicine and Experimental Surgery, Medical University of Lodz, 90-136 Lodz, Poland; zaneta.kaluzinska@umed.lodz.pl (Ż.K.-K.); damian.kolat@umed.lodz.pl (D.K.); 7 Department of Functional Genomics, Medical University of Lodz, 90-752 Lodz, Poland; 8 Department of Internal Medicine and Geriatric Cardiology, Medical Centre for Postgraduate Education, 00-416 Warsaw, Poland; michalchudzik@wp.pl; 9 Department of Nephrology, Hypertension and Family Medicine, Medical University of Lodz, 90-549 Lodz, Poland

**Keywords:** predictors, cardiovascular disease, SARS-CoV-2, COVID-19, long COVID, non-hospitalized

## Abstract

**Background and aims:** Long COVID symptoms persist globally, with a notable rise in cardiovascular disease (CVD) among COVID-19 survivors, including those without prior risk factors or hospitalizations. This study aims to identify predictors of cardiovascular-related Long COVID symptoms. **Methods:** This study included subjects with post-SARS-CoV-2 infections, categorizing them into three groups: non-Long COVID (non-LC), Long COVID (LC), and Long COVID with cardiovascular symptoms (cardio-LC) as part of the Polish Long COVID Cardiovascular (PoLoCOV-CVD) study collected between the years 2020 and 2022, comprising 4000 participants. Chi-square tests and logistic regression were used to report the prevalence and determinants of quality of life in cardio-LC, based on patient self-reported data including comorbidities and medications. **Results:** Of the 704 patients analyzed, 71.9% were female with a median age of 54 years (IQR: 45–64). Cardio-LC patients had statistically significant differences relative to the non-LC group in terms of blood pressure, elevated LDL cholesterol (*p* = 0.010), and non-HDL cholesterol (*p* = 0.013). In addition, cardio-LC patients were more likely to be female (*p* < 0.001) who exhibited psychological conditions, such as sleep disturbances (*p* < 0.001), anxiety (*p* < 0.001), and depression (*p* < 0.001) compared to the non-LC group. However, the multivariable logistic regression analysis revealed that only the female gender and sleep disturbances remained an independent predictor associated with cardio-LC (OR: 1.66, CI 1.12–2.46; OR: 1.742, CI 1.12–2.70) compared to participants without Long COVID. **Conclusions:** The significant positive association of female gender and sleep disturbances with cardiovascular complications during Long COVID highlights critical demographic and psychological factors that deserve attention in clinical practice.

## 1. Introduction

Coronavirus Disease 2019 (COVID-19) primarily affects the respiratory tract and leads to unexpected cases of pneumonia that are associated with elevated body temperature, dry cough, shortness of breath, and hypoxia. While, in particular, clinical imaging revealed interstitial pneumonia on chest X-ray and computed tomography scans [1], numerous studied revealed that it is a multi-organ disease that affects the digestive, cardiovascular, nervous, and immune systems [2,3,4]. Despite the gradual decline of COVID-19, scientists continue to grapple with understanding and addressing its long-term consequence. Recently, patients reporting persistent symptoms after the acute phase of COVID-19 referred to as Long COVID or post-COVID-19 syndrome [5].

However, the pathophysiology of Long COVID remains unclear to date, partly due to the various symptoms and affected organ systems. So far, the gathered evidence listed clinical symptoms such as shortness of breath, fatigue with or without exertion, myalgia, glucose intolerance, inflammatory syndrome, postural orthostatic tachycardia, and peripheral neuropathy, indicative of involvement across multiple organ systems [6,7] in the affected population. Therefore, the terminology used to describe Long COVID symptoms can be confusing due to the complexity and overlap of clinical presentations [8]. This ambiguity hinders and challenges healthcare providers in ensuring an accurate diagnosis and treatment plan for this special group.

A recent analysis of 153,760 patients led by the US Department of Veterans Affairs found a significant increase in the incidence of cardiovascular disease (CVD) in patients who survived COVID-19. After the first 30 days after SARS-CoV-2 infection, patients showed increased cardiovascular risk and 12-month burdens of incident cardiovascular diseases, including cerebrovascular disorders, dysrhythmias, inflammatory heart disease, ischemic heart disease, heart failure, thromboembolic disease, and other cardiac disorders. Cardiovascular risk was still prevalent regardless of age, race, gender, and other cardiovascular risk factors, including obesity, hypertension, diabetes, chronic kidney disease, and hyperlipidemia. Moreover, increased cardiovascular risk was observed even in non-hospitalized patients, which was related to the severity of the acute disease phase [9,10].

Patients with Long COVID may suffer from a variety of symptoms, and understanding the predictors of cardiovascular involvement in Long COVID is evolving [11,12,13]. We decided to study a Polish cohort in this study because there is a lack of sufficient studies exploring the long-term cardiovascular consequences of Long COVID in specific demographic groups. Further research is needed to establish factors or predictors, which could clearly elucidate disease manifestation for an appropriate therapeutic approach in this group of patients. Therefore, our current study aimed to assess the predictors of Long COVID with cardiovascular symptoms.

## 2. Materials and Methods

### 2.1. Study Population

**Figure 1:**
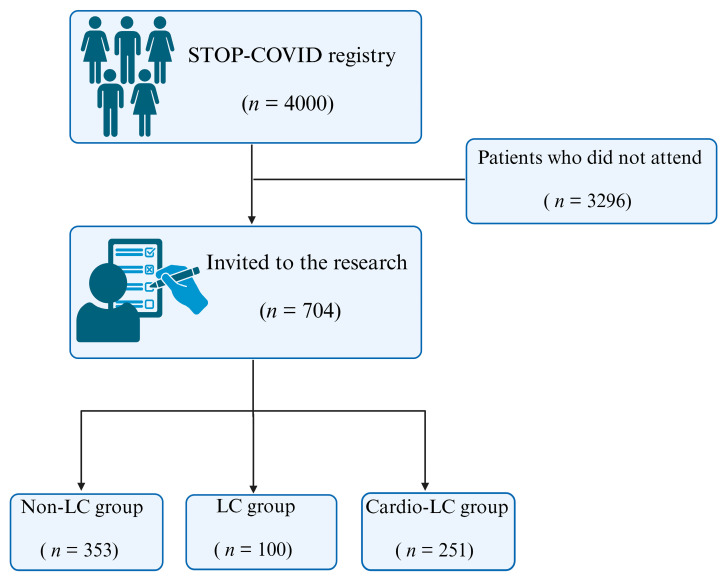
Study flowchart.

The patients included in the Polish Long COVID Cardiovascular (PoLoCOV-CVD) study are a subset of the STOP-COVID registry (ClinicalTrials.gov identifier—NCT05018052). In this retrospective cross-sectional study, nearly 4000 patients were recruited into the registry from 2020 to 2022. Of these, 704 patients who met all the inclusion criteria were assessed in two outpatient clinics and invited to participate in the research (Figure 1). This study included subjects with: (1) laboratory confirmed diagnosis of COVID-19; (2) age ≥18 years; (3) full recovery of acute clinical symptoms, <15 days after the last symptoms, for the non-LC group; (4) written consent to participate in this study; (5) no diagnosed cardiovascular disease; (6) treatment of COVID-19 in the outpatient setting. The exclusion criteria were as follows: (1) lack of written consent from the patient to participate in the study; (2) previously diagnosed cardiovascular disease; (3) hospitalization for COVID-19 disease; (4) high 24 h ambulatory blood pressure, i.e., >130/80 mmHg; (5) anemia; (6) hyperthyroidism or hypothyroidism; (7) history of cancer; (8) treatment with drugs that may have had a cardiotoxic effect; (9) other reversible factors that could lead to decreased left ventricle ejection fraction (LVEF) and/or contractility disorders.

Long COVID was diagnosed according to the WHO definition [14], while the non-cardiovascular symptoms that we chose allowed us to group the non-LC group based on if a patient showed any of the following symptoms: memory and concentration disturbances, headache, excessive sweating, hair loss, muscle pain, anosmia and ageusia, cough, skin lesions, conjunctivitis, arthralgia, and myalgia. On the other hand, the “cardio-LC” criteria were defined by the presence of newly reported cardiovascular-related symptoms such as fatigue, dyspnea, heart palpitations, syncope, chest pain, precordial discomfort, and asthenia [15]. The study participants were divided into three groups:

1. Non-LC group: Individuals who recovered from SARS-CoV-2 infection without experiencing any post-recovery symptoms (*n* = 353);
2. LC group: Individuals diagnosed with Long COVID who did not exhibit cardiovascular symptoms (*n* = 100);
3. Cardio-LC group: Individuals diagnosed with Long COVID who experienced cardiovascular symptoms (*n* = 251).

All patients underwent a comprehensive assessment evaluating their clinical history and physical examination. Subsequently, detailed information on COVID-19 symptoms was collected, which consist of the presence or absence of symptoms and other specific characteristics. These symptoms were documented during the patient’s follow-up visit three months after the diagnosis of COVID-19. Each patient completed a questionnaire regarding persistent symptoms after COVID-19. The collected questionnaire data about the clinical symptoms identified Long COVID as the most common, with ailments occurring four weeks after SARS-CoV-2 infection [16].

### 2.2. Study Variables

We included the following covariates in our analysis: gender, age, body mass index (BMI), total cholesterol, high-density lipoprotein cholesterol (HDL-C), low-density lipoprotein cholesterol (LDL-C), non-HDL cholesterol, triglycerides (TG), fasting glucose, 24 h ambulatory blood pressure monitoring (ABPM) for (including measurements: 24 h systolic BP, 24 h diastolic BP, day activity systolic BP, day activity diastolic BP, night-time systolic BP, night-time diastolic BP), hypertension (yes/no), diabetes (yes/no), and hyperlipidemia (yes/no). Furthermore, insulin tolerance was calculated using the Homeostatic Model Assessment for Insulin Resistance (HOMA-IR) as follows: (1) fasting glucose [mmol/L] × fasting insulin [μmol/L]/22.5] and (2) fasting glucose [mg/dL] × fasting insulin [μmol/L]/405 [17,18]. The variables relating to mental conditions, such as sleep disturbances (yes/no; defined as either difficulty of initiating or maintaining sleep or presence of restless, disturbed nights), anxiety (yes/no), and depression (yes/no), were collected using the latest standard questionnaire provided by the European Society of Cardiology (ESC) [19]. Moreover, the prediction model SCORE2 was studied to estimate 10-year fatal and non-fatal cardiovascular disease risk in European individuals aged 40–69 years without previous CVD [20]. Medical history of venous thromboembolism, asthma, chronic obstructive pulmonary disease, thyroid disease, and comorbidities were obtained from outpatient clinics registered in the patients’ hospital registry. Data on medication taken by each patient were collected during patient interviews, which included angiotensin-converting-enzyme inhibitors (ACEI; yes/no), angiotensin receptor blockers (ARB; yes/no), beta-blockers (yes/no), calcium blockers (yes/no), diuretics (yes/no), and statins (yes/no).

### 2.3. Statistical Analysis

The normality of continuous variables was assessed using the Shapiro–Wilk test. Continuous variables were evaluated using the Mann–Whitney U test and presented as median with the 25–75th percentile. Categorical variables were compared between groups using the chi-square test and presented in percentages. Univariable and multivariable logistic regression analyses were conducted to identify predictors of cardiovascular symptoms in patients with Long COVID. Subsequently, variables that showed statistical significance in the univariable model were included in the multivariable model for covariate adjustments. The results with a *p*-value of less than 0.05 were considered statistically significant. IBM SPSS Statistics software version 25 was used for all analyses (IBM, Armonk, NY, USA).

## 3. Results

### 3.1. Characteristics of the Study Group

The majority of the study participants were females (71.9%). The median age of the patients at the time of their outpatient clinic visit was 54 years (IQR: 45–64). In terms of comorbidities, 39.8% had hypertension, 10.2% had diabetes, 21.6% had hyperlipidemia, 12.1% were smokers, 11.1% had asthma, and 1.3% had COPD. Other conditions, such as thromboembolism, were also present in the cohort. Approximately more than one-third of subjects (33.8%) reported experiencing anxiety, while a similar proportion (33.7%) reported symptoms of depression.

### 3.2. Comparison of Patients with and Without Cardiovascular Symptoms Among Patients with Long COVID

Compared to the LC group, cardio-LC patients demonstrated a significantly higher level of LDL cholesterol and a higher prevalence of asthma (Table 1). In the multivariable adjustment model, only asthma remained an independent predictor associated with cardiovascular symptoms during LC (OR: 2.83; CI: 1.06–7.55) (Table 2).

### 3.3. Comparison of Patients with Cardiovascular Symptoms and Patients Without Long-COVID

Patients with cardiovascular symptoms differed from the non-LC group in terms of gender distribution, several blood pressure, total cholesterol, LDL cholesterol, non-HDAL cholesterol, and fasting glucose level (Table 3). Long COVID patients with cardiovascular symptoms also exhibited inferior psychological condition, as visible in sleep disturbances (*p* < 0.001), anxiety (*p* < 0.001), and depression (*p* < 0.001). However, in the adjusted multivariable logistic regression model, female gender and sleep disturbances remained an independent predictor associated with cardiovascular symptoms during LC (OR: 1.742, CI 1.12–2.70) (Table 4).

## 4. Discussion

We observed notable results when comparing various control groups, including those without LC or with LC, to individuals with a history of cardiovascular complications associated with LC. Some differences in cardiovascular risk factor such as blood pressure and cholesterol levels in cardio-LC group was observed. Moreover, psychological distress such as sleep disturbance and female gender was significantly associated with cardio-LC.

These findings align with some studies that report cardiovascular complications among COVID-19 survivors. In the retrospective study with one year of follow-up, Wang et al. noted an increased risk of atrial fibrillation, myocarditis, ischemic heart disease, and heart failure among patients who survived COVID-19. These individuals also had a higher 12-month risk of incidental cardiovascular diseases than non-COVID-19 controls [21]. A similar follow-up duration allowed Xu et al. to observe an elevation in LDL-C, TG, and total cholesterol, as well as a reduction in HDL-C in COVID-19 survivors relative to individuals with no positive COVID-19 tests in the past. The same authors found dyslipidemia to be the greatest among patients who had the most severe infection and were admitted to the intensive care unit [22]. Interestingly, data from our study indicate that all diastolic and systolic measures were lower in cardio-LC group relative to non-LC group, which was accompanied with elevated LDL, non-HDL, and total cholesterol in the former group.

Mental health consequences of Long COVID, such as sleep disturbances, anxiety, and depression, are consistent with a broader understanding of affected quality-of-life implications [23]. Growing evidence suggests a link between insomnia, depression, anxiety, and cardiovascular disease. In the systematic review by Sofi et al. [24], 13 prospective studies were included in their final analysis. The study showed that insomnia determined an increased risk (45%) of developing or dying from cardiovascular disease during the follow-up [25], including 54 observational studies, which assessed the role of depression as an etiological or prognostic factor in coronary heart disease. Significant associations were observed between depression and coronary heart disease. Moreover, in patients with heart failure and depression, an association between potent antidepressants and an increased risk of death was observed [18]. However, anxiety was an even more decisive risk factor for cardiovascular disease in the study by Allgulander [26], determining other known risk factors, such as overweight, a sedentary lifestyle, or the use of psychoactive substances. Therefore, the ESC’s clinical practice guidelines on cardiovascular disease prevention also refer to psychiatric and psychosocial factors [18]. Mental health difficulties were reported in COVID-19 survivors, which may involve physiological and psychological factors [27].

Identifying asthma and sleep disturbances as independent predictors in the current study aligns with the perception of Long COVID as a complex condition with various contributing factors. Asthma has been associated with severe COVID-19 in some studies, and sleep disturbances are recognized as common in the post-acute phase. Lee et al. admitted that there are higher odds of developing new-onset asthma following COVID-19 infection [28]. Patients with asthma who had undergone mild-to-moderate COVID-19 were investigated by Kwok et al., revealing a relation between infection and a higher need for maintenance therapy due to more uncontrolled asthma [29]. Dolby et al. concluded that patients with poorly controlled or severe asthma had a greater risk of hospitalization due to COVID-19 relative to those without asthma, but this does not apply when investigating mild or well-controlled asthma [30]. It should be noted that even though sleep disturbances had lower OR than asthma, the symptoms of the latter overlap with the long-term effects of COVID-19 [31]. As for sleep disturbances, an online survey prepared by Tedjasukmana et al. showed that nearly 80% of post-COVID patients from various countries confirm having insomnia, sleep-disordered breathing, hypersomnolence, circadian rhythm sleep–wake disorders, parasomnias, and sleep-related movement disorders. The same authors stated that these disturbances typically manifest between 2 and 48 weeks after hospital discharge or negative COVID-19 test but may also last longer [32]. Pena-Orbea et al. indicated that the prevalence of sleep disturbances during post-acute sequelae of COVID-19 ranges between 34% and 50% [33]. This complements the Peng et al.’s research, in which 60% of COVID-19 survivors experienced mental distress even a year after discharge, with daytime dysfunction and loss of energy being among the symptoms that affected quality-of-life to the greatest extent [34]. Sleep disturbances are known to be related to daytime functioning impairment, rendering tiredness [35]. Lastly, a longitudinal study by Tracy et al. evaluated two observation times (one month and three months after discharge) and found that depression in earlier timepoint highly correlated with sleep disturbance but also depression, anxiety, fatigue, cognitive function, and satisfaction with social roles, which manifested in follow-up after three months [36]. If COVID-19 survivors have symptoms overlapping with cardio-LC group, perhaps it may be a manifestation of cardiovascular disease—further diagnosis can be considered among such patients if sleep disturbances occur. CVD and sleep disorders are related in the general population [37]. Indeed, future studies could focus on the mechanisms linking sleep disturbances with the CV symptoms of Long COVID. Although the sympathetic nervous system activity may mediate the association, other mechanisms are also possible and should be described. In addition, it is still not clear if sleep disturbances increase the CV risk in patients suffering from Long COVID. The research on the CV symptoms of Long COVID and CV risk is also warranted, especially focusing on the gender differences. Indeed, although females generally have lower CV risk compared to males, it is not known if this difference is still present if CV symptoms occur following COVID-19 disease. 

This study possesses several limitations. First, subjects or patients enrolled in the study based on self-voluntary reporting COVID-19 status to the medical facility. Consequently, we are unable to include those who did not visit the medical facility. Second, we did not assess flu and COVID-19 vaccination status in the enrolled subjects. Third, this study was conducted on a homogeneous population with a small sample size, which may have contributed to certain factors not reaching statistical significance, despite observable effects. Nonetheless, the evidence presented are from one of the largest Polish registries of patients infected with the SARS-CoV-2 virus (i.e., STOP-COVID registry) data, where the majority of patients received outpatient treatment for COVID-19, which allowed for further follow up. Our statistical approach to compare cardio-long-affected patients with multiple controls greatly enhances inference of the study findings.

## 5. Conclusions

This study highlights the multifaceted nature of cardiovascular complication in Long COVID. Demographic factors, such as female gender, and psychological factors, including sleep disturbances and asthma, significantly contribute to cardiovascular complications. In addition, females have a higher risk of developing Long COVID, which may aid in planning out further steps. These results underline the importance of addressing psychological aspects in the management and prevention of Long COVID-related cardiovascular symptoms.

## Figures and Tables

**Table 1:** Differences in baseline characteristics, clinical assessment, and treatment profile between Long COVID patients with and without cardiovascular symptoms.

Variable		LC Group (*n* = 100)	Cardio-LC Group (*n* = 251)	*p*-Values
Age [years]		54 (46–64)	53 (45–64)	0.501
Gender	Male	20 (20.0)	58 (23.1)	0.527
	Female	80 (80.0)	193 (76.9)	
BMI [kg/m^2^]		26.68 (24.21–29.72)	26.77 (23.52–30.38)	0.908
Hypertension		38 (38.0)	92 (36.7)	0.814
Diabetes		9 (9.0)	29 (11.6)	0.487
Hyperlipidemia		27 (27.0)	48 (19.1)	0.104
Venous thromboembolism		2 (2.0)	0 (0.0)	0.066
Asthma		5 (5.0)	31 (12.4)	0.040
Chronic obstructive pulmonary disease		2 (2.0)	2 (0.8)	0.338
Thyroid disease		18 (18.0)	50 (19.9)	0.681
Any comorbidity		33 (33.0)	94 (37.5)	0.434
Sleep disturbances		81 (82.7)	206 (82.7)	0.986
Anxiety score		2.0 (1.0–3.75)	2.0 (1.0–4.0)	0.809
Depression score		2.0 (1.0–3.0)	2.0 (1.0–4.0)	0.571
Systolic 24 h [mmHg]		124.9 (117.0–127.9)	124.0 (113.0–124.0)	0.265
Diastolic, 24 h [mmHg]		74.6 (69.3–78.0)	73.7 (68.2–77.8)	0.519
Systolic, day [mmHg]		129.8 (123.3–132.9)	129.50 (118.0–133.0)	0.354
Diastolic, day [mmHg]		78.4 (72.7–82.1)	78.0 (72.0–81.9)	0.595
Systolic, night [mmHg]		113.8 (103.9–118.0)	113.8 (102.60–119.0)	0.886
Diastolic, night [mmHg]		66.0 (60.0–68.8)	65.2 (60.0–69.0)	0.801
Total cholesterol [mmol/L]		11.2 (9.6–11.6)	11.2 (10.0–12.4)	0.062
HDL cholesterol [mmol/L]		3.2 2.7–3.6)	3.2 (2.8–3.5)	0.634
LDL cholesterol [mmol/L]		6.6 (5.3–6.9)	6.7 (5.9–8.0)	0.027
Triglicerides [mmol/L]		6.4 (4.6–18.3)	6.4 (4.5–7.3)	0.154
Non-HDL cholesterol [mmol/L]		7.9 (6.6–8.3)	7.9 (6.9–9.1)	0.127
Glucose [mmol/L]		5.4 (5.0–5.6)	5.3 (4.9–5.6–100.5)	0.510
Insulin resistance [mU/mL]		2.27 (1.41–2.90)	2.18 (1.33–2.42)	0.212
ESC SCORE2		3.9 (1.7–7.4)	3.4 (1.2–6.5)	0.356
ACEI		15 (16.3)	40 (18.3)	0.667
ARB		16 (17.4)	34 (15.6)	0.695
Beta-blocker		34 (37.0)	70 (32.1)	0.409
Calcium blocker		10 (10.9)	27 (12.4)	0.707
Diuretic		13 (14.1)	24 (11.0)	0.439
Statin		15 (16.3)	25 (11.5)	0.246

Categorical data are presented as numbers (percentages) and continuous data as median (25–75th). *p*-values are reported as obtained after excluding missing values; the chi-square test was performed for categorical data and Mann–Whitney for continuous data. Abbreviation: LC group—patients diagnosed with Long COVID but without cardiovascular symptoms, Cardio-LC group—patients with confirmed cardiovascular symptoms during Long COVID, BMI—body mass index, HDL—high-density lipoprotein, LDL—low-density lipoprotein, ESC—European Society of Cardiology, SCORE2—Systematic Coronary Risk Evaluation 2, ACEI—angiotensin-converting enzyme inhibitors, ARB—angiotensin receptor blockers.

**Table 2:** Results of the logistic regression analysis of the occurrence of cardiovascular symptoms among the patients with Long COVID.

Variable	Univariable, OR (95% CI)	Multivariable, OR (95% CI) ^a^	Multivariable, OR (95% CI) ^b^
Age [years]	0.99 (0.97–1.01)	0.99 (0.97–1.01)	0.99 (0.97–1.01)
Female gender	0.83 (0.47–1.47)	0.83 (0.47–1.47)	0.65 (0.35–1.20)
BMI [kg/m^2^]	1.00 (0.96–1.05)	1.00 (0.96–1.05)	1.02 (0.98–1.08)
Hypertension	0.94 (0.58–1.52)	1.00 (0.58–1.71)	1.13 (0.63–2.03)
Diabetes	1.32 (0.60–2.90)	1.35 (0.61–3.00)	1.96 (0.80–4.76)
Hyperlipidemia	0.63 (0.37–1.09)	0.66 (0.38–1.16)	0.63 (0.35–1.12)
Asthma	2.67 (1.01–7.09)	2.71 (1.02–7.21)	2.80 (1.04–7.53)
COPD	0.39 (0.05–2.83)	0.40 (0.05–2.98)	0.32 (0.04–2.62)
Thyroid disease	1.13 (0.62–2.05)	1.22 (0.66–2.25)	1.28 (0.68–2.40)
Any comorbidities	1.21 (0.74–1.98)	1.14 (0.67–1.94)	0.79 (0.44–1.40)
Sleep disturbances	1.00 (0.54–1.86)	1.04 (0.56–1.95)	1.25 (0.66–2.38)
Anxiety	0.98 (0.86–1.11)	0.98 (0.86–1.11)	0.92 (0.56–1.49)
Depression	1.01 (0.88–1.16)	1.01 (0.88–1.16)	1.34 (0.82–2.20)
Systolic 24 h [mmHg]	0.99 (0.97–1.00)	0.99 (0.97–1.01)	0.99 (0.97–1.00)
Diastolic, 24 h [mmHg]	0.99 (0.96–1.02)	0.98 (0.96–1.01)	0.99 (0.96–1.02)
Systolic, day [mmHg]	0.99 (0.97–1.01)	0.99 (0.98–1.01)	0.99 (0.97–1.01)
Diastolic, day [mmHg]	0.99 (0.96–1.02)	0.99 (0.96–1.02)	0.99 (0.96–1.02)
Systolic, night [mmHg]	1.00 (0.98–1.01)	1.00 (0.98–1.01)	0.99 (0.98–1.01)
Diastolic, night [mmHg]	1.00 (0.97–1.03)	1.00 (0.97–1.03)	0.99 (0.967–1.03)
Total cholesterol [mmol/L]	1.00 (0.99–1.01)	1.00 (0.99–1.01)	0.79 (0.55–1.12)
HDL cholesterol [mmol/L]	1.00 (0.98–1.02)	1.01 (0.98–1.03)	0.79 (0.46–1.37)
LDL cholesterol [mmol/L]	1.00 (0.99–1.01)	1.006 (1.00–1.01)	1.01 (1.00–1.01)
Tryglicerides [mmol/L]	0.99 (0.99–1.00)	0.99 (0.99–0.99)	0.89 (0.74–1.07)
Non-HDL cholesterol [mmol/L]	1.00 (0.99–1.01)	1.00 (0.99–1.01)	0.99 (0.97–1.01)
Glucose [mmol/L]	0.99 (0.98–1.00)	0.99 (0.98–1.00)	1.00 (0.99–1.01)
Insulin resistance [mU/mL]	0.88 (0.77–1.00)	0.85 (0.74–0.99)	0.84 (0.72–0.99)
ESC SCORE2	0.98 (0.94–1.03)	1.01 (0.93–1.09)	1.02 (0.94–1.11)
ACEI	1.15 (0.60–2.21)	1.21 (0.61–2.40)	1.14 (0.57–2.29)
ARB	0.87 (0.45–1.68)	0.90 (0.46–1.77)	0.94 (0.46–1.92)
Beta-blocker	0.80 (0.48–1.34)	0.82 (0.48–1.39)	0.89 (0.51–1.56)
Calcium blocker	1.15 (0.53–2.50)	1.22 (0.55–2.72)	1.27 (0.57–2.85)
Diuretics	0.75 (0.36–1.55)	0.76 (0.36–1.59)	0.79 (0.36–1.71)
Statin	0.66 (0.33–1.32)	0.66 (0.32–1.36)	0.73 (0.35–1.52)

^a^ Adjusted for age and gender; ^b^ Adjusted for age, gender, BMI, LDL cholesterol, insulin resistance and asthma. Abbreviation: OR—odds ratio, CI—confidence interval, BMI—body mass index, HDL—high-density lipoprotein, LDL—low-density lipoprotein, TG—triglycerides, COPD—chronic obstructive pulmonary disease, ESC—European Society of Cardiology, SCORE2—Systematic Coronary Risk Evaluation 2, ACEI—angiotensin-converting enzyme inhibitors, ARB—angiotensin receptor blockers.

**Table 3:** Differences in baseline characteristics, clinical assessment, and treatment profile between subjects with no symptoms after recovery from SARS-CoV-2 infection and Long COVID patients with cardiovascular symptoms.

Variables		Non-LC Group (*n* = 353)	Cardio-LC Group (*n* = 251)	*p*-Values
Age [years]		54 (44–65)	53 (45–64)	0.850
Gender	Male	120 (34.0)	58 (23.1)	0.004
	Female	233 (66.0)	193 (76.9)	
BMI [kg/m^2^]		27.2 (24.0–30.5)	26.8 (23.5–30.4)	0.465
Hypertension		150 (42.5)	92 (36.7)	0.149
Diabetes		34 (9.6)	29 (11.6)	0.446
Hyperlipidemia		77 (21.8)	48 (19.1)	0.421
Venous thromboembolism		1 (0.3)	0 (0.0)	0.347
Asthma		42 (11.9)	31 (12.4)	0.866
Chronic obstructive pulmonary disease		5 (1.4)	2 (0.8)	0.483
Thyroid disease		61 (17.3)	50 (19.9)	0.409
Any comorbidity		244 (69.1)	157 (62.5)	0.092
Sleep disturbances		238 (67.6)	206 (82.7)	<0.001
Anxiety score		2.0 (0.0–3.0)	2.0 (1.0–4.0)	<0.001
Depression score		2.0 (0.0–3.0)	2.0 (1.0–4.0)	<0.001
Systolic 24 h [mmHg]		124.93 (117.0–132.85)	124.0 (113.0–128.0)	0.005
Diastolic, 24 h [mmHg]		74.56 (70.40–80.90)	73.70 (68.20–77.80)	0.001
Systolic, day [mmHg]		129.76 (123.00–139.20)	129.50 (118.0–133.0)	0.007
Diastolic, day [mmHg]		78.40 (74.80–84.05)	78.0 (72.0–81.90)	0.002
Systolic, night [mmHg]		113.78 (106.0–121.95)	113.78 (102.60–119.0)	0.028
Diastolic, night [mmHg]		65.96 (61.95–71.55)	65.20 (60.0–69.0)	0.008
Total cholesterol [mmol/L]		11.17 (9.77–12.0)	11.17 (9.99–12.43)	0.043
HDL cholesterol [mmol/L]		3.22 (2.83–3.66)	3.22 (2.83–3.55)	0.478
LDL cholesterol [mmol/L]		6.7 (5.3–7.3)	6.7 (5.8–8.0)	0.010
Triglicerides [mmol/L]		6.2 (4.2–6.9)	6.4 (4.4–7.3)	0.227
Non-HDL cholesterol [mmol/L]		7.9 (6.5–8.3)	7.9 (6.9–9.2)	0.013
Insulin resistance [mU/mL]		2.1 (1.2–2.3)	2.2 (1.3–2.4)	0.175
Glucose [mmol/L]		5.6 (5.1–5.8)	5.3 (4.9–5.6)	0.001
ESC SCORE2		4.0 (1.4–8.7)	3.4 (1.2–6.5)	0.135
ACEI		44 (14.1)	40 (18.3)	0.193
ARB		44 (14.1)	34 (15.6)	0.644
Beta-blocker		101 (32.5)	70 (32.1)	0.929
Calcium blocker		31 (10.0)	27 (12.4)	0.381
Diuretic		45 (14.5)	24 (11.0)	0.245
Statin		40 (12.9)	25 (11.5)	0.631

Categorical data are presented as numbers (percentages) and continuous data as median (25–75th). *p*-values are reported as obtained after the exclusion of missing values. Abbreviations: Non-LC group—patients with no symptoms after recovery from SARS-CoV-2 infection, Cardio-LC group—patients with confirmed cardiovascular symptoms during Long COVID, OR—odds ratio, CI—confidence interval, BMI—body mass index, HDL—high-density lipoprotein, LDL—low-density lipoprotein, ESC—European Society of Cardiology, SCORE2—Systematic Coronary Risk Evaluation 2, ACEI—angiotensin-converting enzyme inhibitors, ARB—angiotensin receptor blockers.

**Table 4:** Results of the logistic regression analysis of the occurrence of cardiovascular symptoms compared to the patients without Long COVID.

Variables	Univariable, OR (95% CI)	Multivariable, OR (95% CI) ^a^	Multivariable, OR (95% CI) ^b^
Age [years]	0.99 (0.98–1.00)	0.99 (0.98–1.00)	0.99 (0.97–1.01)
Female	1.71 (1.18–2.47)	1.71 (1.18–2.47)	1.66 (1.12–2.46)
BMI [kg/m^2^]	0.99 (0.96–1.02)	1.00 (0.97–1.03)	1.00 (0.97–1.03)
Hypertension	0.78 (0.56–1.09)	0.84 (0.58–1.22)	0.76 (0.49–1.17)
Diabetes	1.22 (0.72–2.07)	1.29 (0.75–2.22)	1.40 (0.78–2.52)
Hyperlipidemia	0.84 (0.56–1.26)	0.83 (0.55–1.25)	0.73 (0.47–1.11)
Asthma	1.04 (0.63–1.71)	1.02 (0.62–1.68)	0.99 (0.59–1.65)
Chronic obstructive pulmonary disease	0.55 (0.10–2.90)	0.72 (0.13–3.85)	0.89 (0.16–5.02)
Thyroid disease	1.19 (0.78–1.80)	1.05 (0.68–1.61)	1.06 (0.68–1.65)
Any comorbidities	0.74 (0.53–1.05)	0.74 (0.51–1.06)	0.68 (0.46–1.01)
Sleep disturbances	2.29 (1.54–3.41)	2.18 (1.46–3.26)	1.87 (1.23–2.85)
Anxiety	1.18 (1.08–1.30)	1.17 (1.06–1.28)	1.21 (0.80–1.83)
Depression	1.20 (1.09–1.32)	1.18 (1.07–1.30)	1.30 (0.86–1.95)
Systolic 24 h [mmHg]	0.98 (0.97–0.99)	0.98 (0.97–1.00)	0.99 (0.97–0.99)
Diastolic, 24 h [mmHg]	0.96 (0.94–0.98)	0.96 (0.94–0.99)	0.97 (0.95–0.99)
Systolic, day [mmHg]	0.98 (0.97–1.00)	0.99 (0.98–1.00)	0.99 (0.98–1.02)
Diastolic, day [mmHg]	0.96 (0.94–0.98)	0.97 (0.95–0.99)	0.97 (0.95–0.99)
Systolic, night [mmHg]	0.98 (0.97–1.00)	0.99 (0.98–1.00)	0.99 (0.98–1.01)
Diastolic, night [mmHg]	0.97 (0.95–0.99)	0.97 (0.95–0.99)	0.98 (0.96–1.00)
Total cholesterol [mmol/L]	1.08 (1.00–1.168)	1.08 (1.00–1.17)	1.00 (1.00–1.01)
HDL cholesterol [mmol/L]	0.87 (0.68–1.11)	0.73 (0.56–0.96)	0.98 (0.96–0.99)
LDL cholesterol [mmol/L]	1.12 (1.03–1.22)	1.13 (1.04–1.23)	1.12 (1.02–1.22)
Triglicerides [mmol/L]	1.01 (0.96–1.07)	1.03 (0.98–1.09)	1.00 (0.99–1.01)
Non-HDL cholesterol [mmol/L]	1.11 (1.02–1.20)	1.11 (1.03–1.21)	1.01 (1.00–1.01)
Insulin resistance [mU/mL]	1.03 (0.92–1.16)	1.090 (0.96–1.23)	1.09 (0.96–1.25)
Glucose [mmol/L]	0.99 (0.98–1.00)	0.94 (0.800–1.11)	0.93 (0.78–1.10)
ESC SCORE2	0.97 (0.94–1.00)	0.97 (0.92–1.01)	0.97 (0.92–1.02)
ACEI	1.36 (0.85–2.17)	1.65 (1.01–2.71)	1.60 (0.97–2.64)
ARB	1.12 (0.69–1.82)	1.18 (0.71–1.94)	1.37 (0.81–2.32)
Beta-blocker	0.98 (0.67–1.42)	1.05 (0.71–1.54)	0.96 (0.64–1.43)
Calcium blocker	1.27 (0.73–2.20)	1.41 (0.80–2.49)	1.20 (0.65–2.22)
Diuretics	0.73 (0.43–1.24)	0.80 (0.46–1.37)	0.79 (0.45–1.41)
Statin	0.87 (0.51–1.49)	0.97 (0.56–1.69)	1.14 (0.63–2.04)

^a^ Adjusted for age and gender. ^b^ Adjusted for age, gender, BMI, LDL, sleep disturbance, depression, anxiety, and ACEI. Abbreviation: OR—odds ratio, CI—confidence interval, BMI—body mass index, HDL—high-density lipoprotein, LDL—low-density lipoprotein, ESC—European Society of Cardiology, SCORE2—Systematic Coronary Risk Evaluation 2, ACEI—angiotensin-converting enzyme inhibitors, ARB—angiotensin receptor blockers.

## Data Availability

The data presented in this study are available upon request from the corresponding author.
